# Impact of Chestnut and Quebracho Tannins on Rumen Microbiota of Bovines

**DOI:** 10.1155/2017/9610810

**Published:** 2017-12-28

**Authors:** Juan María Díaz Carrasco, Claudio Cabral, Leandro Martín Redondo, Natalia Daniela Pin Viso, Darío Colombatto, Marisa Diana Farber, Mariano Enrique Fernández Miyakawa

**Affiliations:** ^1^Instituto de Patobiología, Centro Nacional de Investigaciones Agropecuarias, Instituto Nacional de Tecnología Agropecuaria, Calle Las Cabañas y Los Reseros s/n, Casilla de Correo 25, Castelar, 1712 Buenos Aires, Argentina; ^2^Consejo Nacional de Investigaciones Científicas y Técnicas, Godoy Cruz 2290, 1425 Buenos Aires, Argentina; ^3^Animal Nutrition, Silvateam, Indunor, Cerrito 1136, 1010 Buenos Aires, Argentina; ^4^Instituto de Biotecnología, Centro Nacional de Investigaciones Agropecuarias, Instituto Nacional de Tecnología Agropecuaria, Calle Las Cabañas y Los Reseros s/n, Casilla de Correo 25, Castelar, 1712 Buenos Aires, Argentina; ^5^Departamento de Producción Animal, Facultad de Agronomía, Universidad de Buenos Aires, Av. San Martín 4453, 1417 Buenos Aires, Argentina

## Abstract

The use of phytogenic dietary additives is being evaluated as a means to improve animal productivity. The effect of tannins seems to be the influence not only directly on the digestive process through binding of dietary proteins but also indirectly over their effects on gastrointestinal microbiota. High-throughput sequencing of 16S rRNA gene was used to analyze the impact of dietary supplementation with a blend of chestnut and quebracho tannins on the rumen microbiota of Holstein steers. Bacterial richness was lower in tannins treated animals, while the overall population structure of rumen microbiota was not significantly disturbed by tannins. The ratio of the phyla Firmicutes and Bacteroidetes, a parameter associated with energy harvesting function, was increased in tannins supplemented animals, essentially due to the selective growth of Ruminococcaceae over members of genus* Prevotella*. Fibrolytic, amylolytic, and ureolytic bacterial communities in the rumen were altered by tannins, while methanogenic archaea were reduced. Furthermore, ruminal pH was significantly higher in animals supplemented with tannins than in the control group, while urease activity exhibited the opposite pattern. Further work is necessary to assess the relation between tannins impact on rumen microbiota and alteration of rumen fermentation parameters associated with bovine performance.

## 1. Introduction

The use of bioactive phytochemicals as natural feed additives has been extensively studied as a strategy to manipulate rumen fermentation in pursuit of an improvement in cattle productivity by reducing methanogenesis and increasing the efficiency of nitrogen utilization [1–3]. However, the lack of sufficient understanding about the rumen microbiome is considered one of the major knowledge gaps that hinder effective enhancement of the rumen function.

Tannins are a complex group of polyphenolic compounds found in many plants species which are commonly included in ruminant diets such as forage and sorghum [4]. Tannins are classified as hydrolysable or condensed based on their chemical structure. The molecular structure of hydrolysable tannins includes a core of glucose esterified with gallic and hexahydroxydiphenic acids. Condensed tannins are usually referred to as proanthocyanidins, polymers of flavon-3-ols or flavon-3, 4-diols such as catechin and epicatechin [5]. Tannins can complex with proteins, starch, vitamins, and minerals at moderate pH, as in ruminal conditions, and dissociate at lower pH, including abomasum and the initial portion of the duodenum [6]. The increased digestibility and efficiency of feed utilization induced by addition of tannins to ruminant diets have been attributed to their ability to precipitate proteins, allowing bypass of ruminal digestion and enhancing protein availability at small intestinal level. However, ruminal bypass cannot entirely explain the performance improvement associated with tannins addition in feed.

For a long time, tannins were thought to reduce weight gain and efficiency of nutrient utilization. However, it is now known that their effect may be either beneficial or detrimental depending on tannins origin, molecular structure, dosage, and animal species [4, 7–9]. High doses of tannins reduce voluntary feed intake and nutrient digestibility, whereas moderate concentrations can improve feed utilization [4]. A blend of tannins extracted from quebracho and chestnut tree has been used as additives to improve performance of ruminants and to reduce urinary nitrogen excretion [10]. A recent report showed that addition of moderate concentrations of chestnut and quebracho tannins to the diets of dairy cows did not affect animal performance but increased milk protein yield and decreased urinary nitrogen excretion [11]. Other authors observed that inclusion of chestnut and quebracho tannins increased dry matter intake, average daily gain, and final body weight of steers during the finishing feedlot phase [12]. These two types of tannins differ in their nutritional role and toxic effects in livestock nutrition.

Tannins modify the digestive processes of ruminants not only by binding dietary protein but also through modulation of rumen microbiota [7, 13]. The bovine rumen houses a complex and highly dense microbiota that is responsible for cattle ability to convert indigestible plant mass into energy. In recent years, a link between gut microbiota composition and energy harvesting function has been observed in humans and mice [14, 15]. Rumen microbiota composition was found to be strongly correlated with daily milk-fat yield in bovines [16, 17]. The microbial populations of the rumen and the variations associated with diet have been previously described [18]. However, although much research has been done regarding the effects of tannins on ruminants physiology and their metabolic fate [19], the impact of chestnut and quebracho tannins on rumen microbiota of bovines has not been fully described. The hypothesis under study in the present work is that tannins alter the bacterial populations of the rumen and therefore can be used as a dietary strategy to modulate rumen function. The aim of this study was to analyze the* in vivo* effects of a tannins blend derived from chestnut and quebracho on rumen bacterial populations of Holstein cattle by means of massive 16S rRNA gene sequencing, exploring the relationship between rumen microbiome composition and physiological parameters.

## 2. Materials and Methods

### 2.1. Animal Handling, Dietary Treatments, and Determination of Rumen Parameters

The study was carried out using ruminally fistulated Holstein cows of the Holando−Argentino breed (*n* = 6) with an average body weight of 584 ± 12 kg (mean ± SD). In order to emulate productive conditions, diet was gradually changed from low starch (60% alfalfa bale, 40% concentrate composed of 80% ground corn grain and 20% soybean meal) to high starch (20% alfalfa bale, 80% concentrate) over an adaptation period of 14 days (from d 1 to d 14). Animals were kept on high starch diet for 21 days (from d 15 to d 35) before the beginning of dietary treatments in order to ensure complete adaptation of rumen microbiota. On day 36, animals were randomly divided into two groups of 3 steers and each group was assigned to one of the two dietary treatments: (1) control group without additives or (2) tannins-supplemented group in which a blend of chestnut and quebracho tannins was added to diet at a concentration of 2 g per kg of feed (Table 1). Control and tannins-supplemented diets were administered to the animals until the end of the treatment period (from d 36 to d 48). The twelve-day treatment period was repeated once, after a “washout” period of 21 days during which all animals received the control diet. Ruminal samples were taken from each animal at the end of each treatment period for microbiota analysis. Diet was offered ad libitum during the whole experiment as a total mixed ration once daily at 0800 h. The average dry matter intake was 12.8 ± 0.6 kg per animal per day (mean ± SD).

The tannins blend was obtained from Silvateam (Indunor SA, Argentina) and contained one-third chestnut wood* (Castanea sativa)* tannins extract and two-thirds quebracho* (Schinopsis lorentzii)* tannins extract by weight. Quebracho extract is mainly composed of flavan-3-ols condensed tannins (>84%) while chestnut extract contains mainly digalloyl glucose hydrolysable tannins (>78%). A detailed description of quebracho and chestnut extracts chemical composition has been described elsewhere [11].

Ruminal contents were collected via a dorsal fistula from the ventral sac of the rumen, taking handfuls of material from the interface between the solid material and liquid layer. Samples were collected in sterile containers (200 mL including solid and liquid material), frozen in liquid nitrogen, and stored at −80°C until further processing. Part of the sample was used for determination of ruminal physiological parameters. The ruminal liquor pH was measured using a standard pH meter. Urease activity (UA) was measured according to the Caskey−Knapp method modified by AACC. Nonprotein nitrogen (NPN) was determined through Kjeldahl method (VELP Scientifica, Italy). Animals were cared for by trained personnel only and the experimental protocol and procedures used were conducted according to protocol 27/2011 of the Institutional Committee for the Care and Use of Experimental Animals (CICVyA-INTA).

### 2.2. DNA Extraction

Twenty milliliters of evenly homogenized ruminal liquor was lyophilized before DNA extraction in order to maximize microbial density (1 g of dry material per sample). DNA extraction was conducted using the QIAamp DNA stool kit (Qiagen, Hilden, Germany) following the instructions of the manufacturer with slight modifications. Briefly, 100 mg of lyophilized rumen was weighed and lysed by incubation for 5 min at 95°C. DNA elution was done with 100 *μ*l of Buffer AE, after incubation for 10 min at room temperature. DNA quality was assessed by agarose gel electrophoresis and DNA concentration was determined using a NanoDrop ND-1000 spectrophotometer (NanoDrop Technologies, Wilmington, DE, USA). DNA was kept at −20°C until further processing.

### 2.3. High-Throughput Sequencing of 16S rRNA Gene

The 16S rRNA gene V3-V4 regions were amplified using Illumina primers (forward: 5′ CCTACGGGNGGCWGCAG 3′, reverse: 5′ GGACTACHVGGGTATCTAATCC 3′) with standard adapter sequences attached for barcoding and multiplexing. 16S gene libraries construction and high-throughput sequencing were performed at Macrogen Inc. (Seoul, South Korea) in the Illumina MiSeq platform following manufacturer's instructions [20]. In order to reduce unbalanced and biased base compositions, 15% of PhiX control library was spiked into the amplicon pool. Due to an issue with the length of reverse reads, which were not long enough to achieve merging of paired-end sequences, only forward reads covering V3 and its flanking regions were used for further bioinformatics analysis. The datasets generated in this study are available under request.

### 2.4. Bioinformatics Analysis

FASTQ files were trimmed using Trimmomatic v0.33 [21], which removed all primer and adapter sequences and also removed leading and trailing bases if quality value was below 9 and 3, respectively. Sliding window trimming was also performed, as well as cutting if the average quality within a 4-base window falls below* Q*15. Demultiplexing and quality filtering were done using the script* split_libraries_fastq.py*, which is part of the QIIME v1.9.1 software package [22]. A threshold of Phred quality score (*Q* > 20) of the base was chosen for stringent quality control processing. Chimeric sequences were filtered out in QIIME using the USERCH algorithm. Open-reference operational taxonomic units (OTUs) picking was performed using UCLUST and USEARCH algorithms in QIIME. Each sequence was assigned taxonomy against Greengenes reference OTU build version 13.8, using a 97% sequence similarity threshold. OTUs with abundance below 0.005% were removed from final OTU table, in order to avoid microbial diversity overestimation [23]. Normalization of OTU counts was done by performing multiple rarefactions from 10.000 to 210.000 sequences with steps of 10.000 and with 10 repetitions at each rarefaction depth. The resulting multiple rarefied OTU tables were used for all further analysis. Principal coordinate analysis (PCoA) plots were generated in QIIME with default options using a distance matrix calculated by unweighted UniFrac metric. The significance of grouping in the PCoA plot was tested by analysis of similarity (ANOSIM) in QIIME with 999 permutations. This work used computational resources from the Bioinformatics Unit, Instituto de Biotecnología (CICVyA-INTA).

### 2.5. Statistical Analysis

Relative abundances of bacterial populations were statistically analyzed using STAMP v2.1.3 [24]. The relative abundances of bacterial taxa in control and tannins treated groups were compared at each level of classification (phylum, class, order, family, and genus) using White's nonparametric two-tailed *t*-test with 1.000 permutations. Comparisons in physiological data and diversity estimators were calculated using nonparametric two-tailed Mann−Whitney test (GraphPad Software, San Diego, CA, USA) and were considered statistically significant if *p* < 0.05.

## 3. Results

After high-throughput sequencing, 3,812,179 reads were obtained with an average of 346,562 ± 42,326 reads per sample. Stringent filtration of the sequences based on length and quality was performed before taxonomy assignation, resulting in 2,951,356 reads with an average length of 267 ± 22 base pairs.

**Figure 1:**
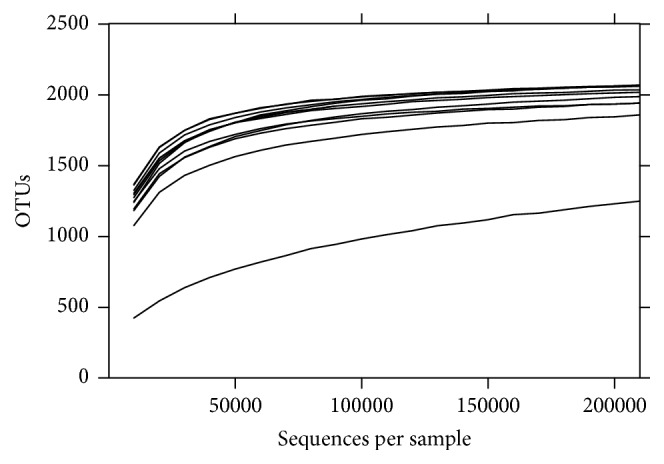
OTUs rarefaction curves of rumen microbiota based on 16S rRNA gene sequences. OTUs were picked using the UCLUST method with 3% dissimilarity in QIIME. Each curve corresponds to a single ruminal sample.

**Figure 2:**
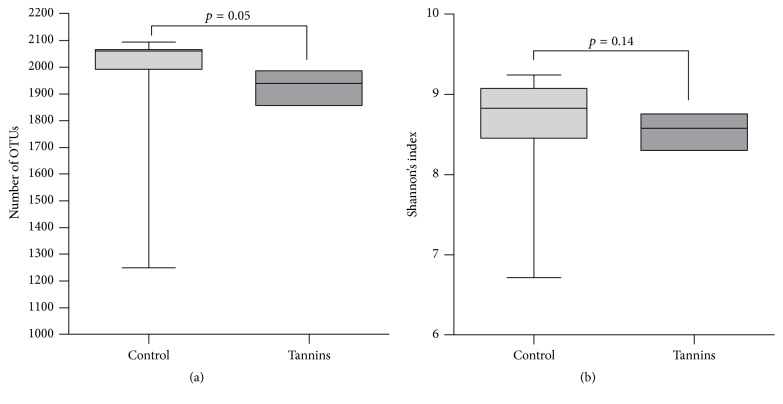
Effect of tannins treatment on (a) bacterial richness (number of OTUs) and (b) Shannon's diversity index of rumen microbiome. Line = median. Box = 25–75 percentiles. Bar = 5–95 percentiles.

**Figure 3:**
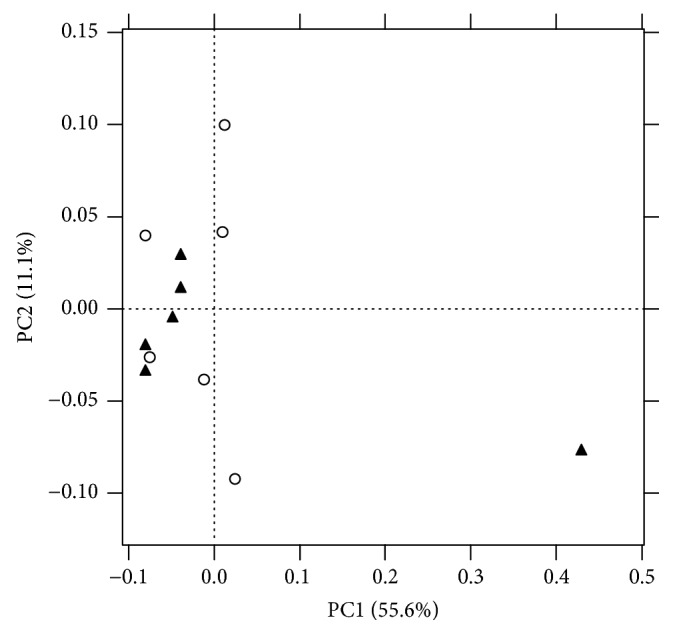
PCoA plot based on unweighted UniFrac metric. Items shaped with triangles and circles correspond to samples from control and tannins treated animals, respectively. Axes (PC1 = 55.6% and PC2 = 11.1%) account for 66.7% of the total variation detected.

The total number of OTUs detected after filtration was 2.263, but this number exhibited a high interindividual variation (Figure 1). We found that the number of OTUs tended to be lower in tannins-supplemented animals than in the control group (*p* = 0.05) (Figure 2(a)). Shannon's diversity index, which estimates the internal sample complexity, was not significantly affected by tannins (*p* = 0.14) (Figure 2(b)). PCoA based on unweighted UniFrac metric was performed in order to explore dissimilarities in microbial composition of the rumen among treated groups (Figure 3). ANOSIM detected no significant differences in bacterial diversity between control and tannins dietary treatments (*p* = 0.86).

**Figure 4:**
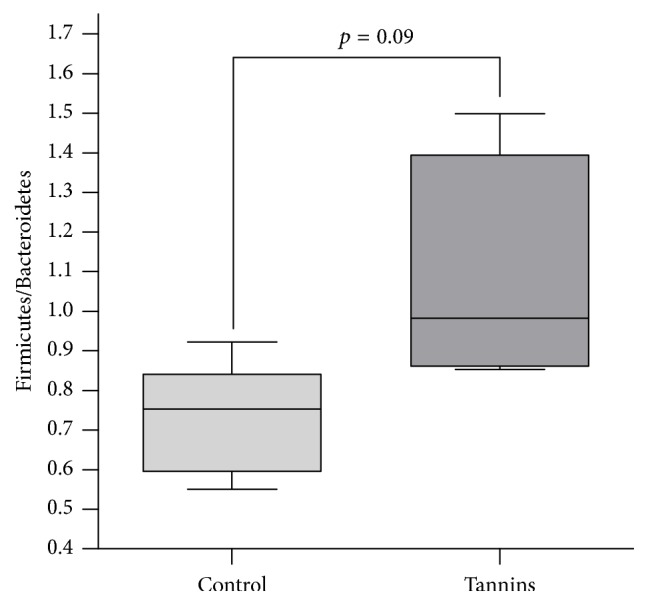
Effect of tannins on the ratio of phyla Firmicutes and Bacteroidetes in rumen microbiota. Line = median. Box = 25–75 percentiles. Bar = 5–95 percentiles.

Firmicutes and Bacteroidetes were the dominant bacterial phyla in the bovine ruminal fluid, accounting for nearly 90% of total microbiota. However, large interindividual variance was observed in the relative abundance of Bacteroidetes and Firmicutes among different animals. In the control group, bacterial populations belonging to phylum Bacteroidetes were the most abundant in all animals (52.1% on average) while Firmicutes accounted for 37.6% of total microbiota. However, this predominance was inverted in the tannins treated animals, with a significantly higher percentage of Firmicutes (46.2%, *p* = 0.02) and a reduction to 44.6% in Bacteroidetes (*p* = 0.18). Accordingly, steers supplemented with tannins presented a trend to higher Firmicutes to Bacteroidetes ratio in comparison with the control group (1.08 versus 0.73, *p* = 0.09) (Figure 4).

**Figure 5:**
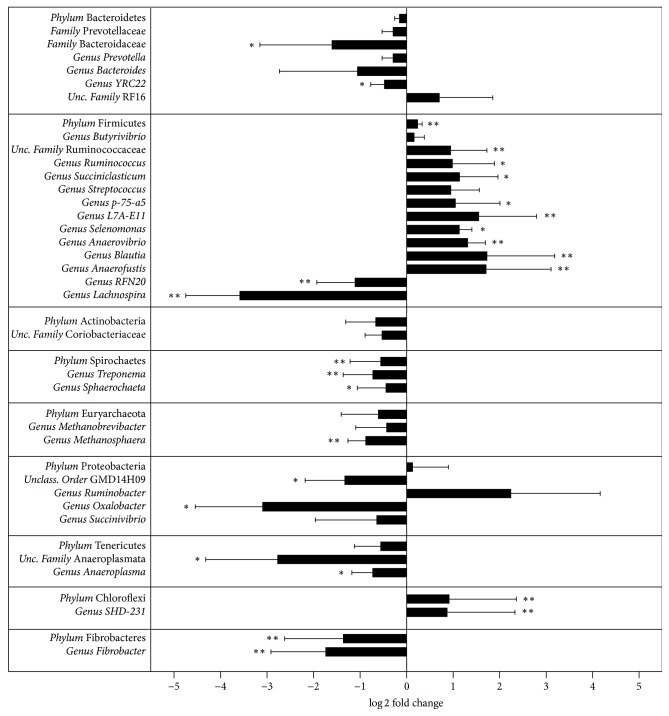
Relative fold changes (log⁡2 tannins/control) in the abundance of rumen bacterial taxa between control and tannins treated steers. ^*∗∗*^*p* < 0.05. ^*∗*^*p* < 0.10. Bar = SEM.

Significant differences in the abundance of certain taxa were detected between control and tannins treated animals (Figure 5). Among Bacteroidetes, the most abundant genus was* Prevotella* accounting for more than 40% of this phylum. The average abundance of* Prevotella* was lower in tannins-supplemented animals than in the control group, although it exhibited a high degree of variance among animals (16.5 versus 21.9%, *p* = 0.15).* Clostridia* was the predominant class which accounted for more than 90% of total Firmicutes, and it was significantly enhanced in tannins treated animals (41.5 versus 34.6, *p* < 0.001). Among* Clostridia*, Ruminococcaceae was the most abundant family and showed a significantly higher abundance in the tannins-supplemented animals (17.8% versus 10.7%, *p* = 0.009). In the control group, most sequences corresponding to family Ruminococcaceae belonged to unclassified members (7.9%) and genus* Ruminococcus* (2.6%). Both taxa were enhanced in tannins treated steers, reaching abundances of 12.6%  (*p* = 0.01) and 4.9% (*p* = 0.07), respectively. Other nonclostridial bacteria within the phylum Firmicutes were significantly altered by tannins, including members of class Erysipelotrichi. Some Erysipelotrichi were enhanced (genus* L7A-E11*, *p* = 0.02; and genus* p-75-A5*, *p* = 0.06) while others were lowered (genus* RFN20*, *p* = 0.001) in tannins*-*supplemented animals. Members of class Bacilli (genera* Streptococcus* and* Lactobacillus*) showed only moderate increases in their abundance. Meanwhile, genus* Fibrobacter *was significantly affected by tannins, accounting for 0.10% of total microbiota in the control group and only 0.005% in the tannins treated animals (*p* = 0.01). Other minor fibrolytic bacteria were significantly more abundant in tannins treated steers, including genus* Blautia* (0.08 versus 0.02%, *p* = 0.01) and member of family Eubacteriaceae genus* Anaerofustis* (0.06 versus 0.02%, *p* = 0.03).

Among sugar fermenting bacteria, the most abundant taxon was genus* Prevotella*, whose abundance was reduced by 5.4% in tannins treated animals, as mentioned above. Genus* Treponema* was also reduced in tannins treated steers (0.41 versus 1.21%, *p* = 0.04). Among Veillonellaceae members, genus* Succiniclasticum*, which specializes in fermenting succinate to propionate, doubled their levels in tannins treated animals (3.99 versus 1.99%, *p* = 0.08). Lipolytic genus* Anaerovibrio* was significantly enhanced by tannins (0.11 versus 0.05%, *p* = 0.01). Genus* Selenomonas* was also increased in tannins supplemented animals (0.11 versus 0.05%, *p* = 0.07). Among ureolytic bacteria, genus* Butyrivibrio* was the most abundant one and it was negatively affected by tannins treatment (1.80 versus 2.36%), as well as* Treponema* and* Succinivibrio* (0.009 versus 0.02%). On the other hand, methanogens belonging to phylum Euryarchaeota were less abundant in tannins supplemented steers (1.37 versus 2.03%) and their levels were inversely correlated with rumen pH (*r* = −0.80). Genus* Methanosphaera* was significantly reduced by tannins (0.06% versus 0.16%, *p* = 0.01).

**Figure 6:**
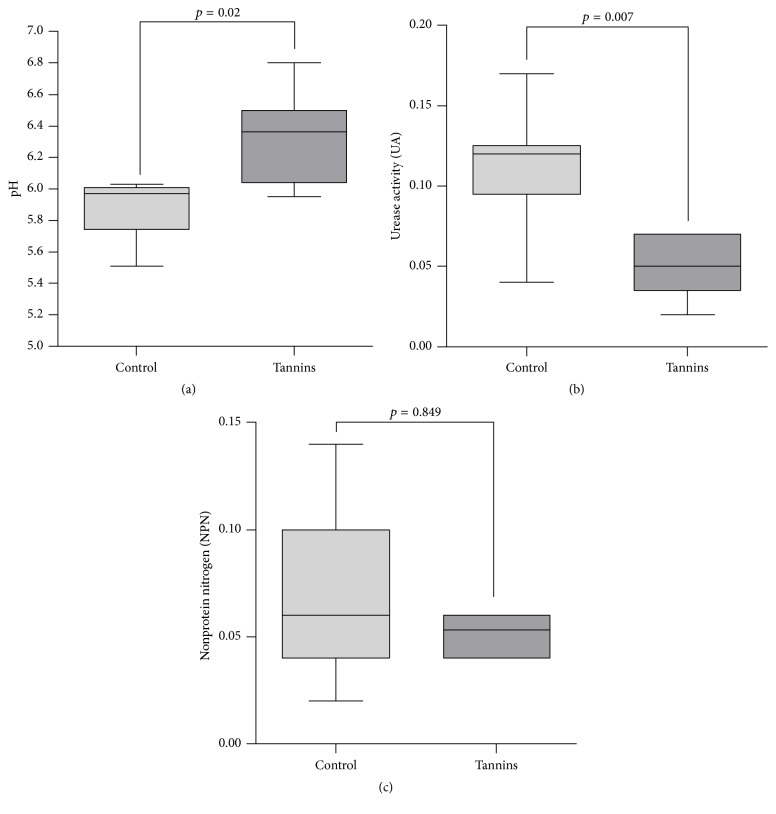
Effect of tannins on pH, urease activity, and NPN in the rumen liquor. Line = median. Box = 25–75 percentiles. Bar = 5–95 percentiles. Urease activity values are given in pH units proportional to urease activity. NPN levels are expressed as a percentage of soluble nitrogen in the rumen.

Determination of pH, urease activity, and NPN was performed in all rumen samples along with microbiota composition analysis. Tannins treated steers had significantly higher ruminal pH than the control group (6.30 versus 5.88, *p* = 0.02) (Figure 6(a)). Urease activity exhibited the opposite pattern, showing a significant decline in the tannins treated steers (Figure 6(b)). Moreover, a strong negative correlation between pH and urease activity was detected (*r* = −0.95). NPN was not significantly altered by treatments (Figure 6(c)).

## 4. Discussion

Rumen microbiome diversity is a key feature of ruminants that confers cattle the ability to adapt to a wide range of dietary conditions [25]. In recent years, the concept of host microbiome individuality in ruminants is gaining support, since numerous studies found a large number of taxa whose presence or abundance in the rumen varies markedly among individuals [26–28]. Dietary tannins diminished ruminal richness but did not significantly affect the bacterial communities' complexity (i.e., balance between the relative abundances of taxa). A recent report found an increase in rumen richness but no change in Shannon's diversity index after supplementation with a blend of polyphenols essential oil in dairy heifers under a high grain diet, supporting the idea that polyphenols can affect bacterial richness without disrupting the overall rumen microbiota population structure [29]. In line with this, *β*-diversity analysis detected no significant differences in rumen bacterial diversity between control and tannins treated steers. Low microbial richness in the rumen has been recently found to be tightly linked to a higher feed efficiency in dairy cows [30]. The authors suggest that lower richness in the rumen of efficient animals results in a simpler metabolic network which leads to higher concentrations of specific components that are used to support the host's energy requirements. Together, diversity analyses suggest that bacterial richness was decreased, while the overall bacterial complexity of the rumen was not significantly affected by chestnut and quebracho tannins supplementation.

The dominance of phyla Firmicutes and Bacteroidetes in the bovine ruminal fluid is a common feature in the gastrointestinal microbiome of monogastric organisms and ruminants [15, 17, 28, 31]. Henderson et al. also found that an increase in the ruminal abundance of total Firmicutes correlated with a decrease in the abundance of Bacteroidetes both in cows (*r* = −0.80) and in sheep (*r* = −0.97) [32]. Other authors found that Bacteroidetes were the most abundant phylum in ruminal samples obtained from dairy cows but some animals exhibited a higher percentage of Firmicutes compensating for a lower abundance of Bacteroidetes [17]. Our results agree with these observations, since a strong inverse correlation between the abundances of Firmicutes and Bacteroidetes was detected (*r* = −0.99). These results suggest that members of Firmicutes and Bacteroidetes compete for available resources in the rumen and tannins would tip the balance in favor of Firmicutes.

The ratio of Firmicutes to Bacteroidetes has been shown to affect energy harvesting and body fat accumulation in humans and mice [14, 15]. Along with increased fatty acid absorption, more energy was found to be efficiently obtained from diet in obese mice, illustrating the connection between Firmicutes and improved efficiency in energy harvesting [14]. In cows, the Firmicutes to Bacteroidetes ratio was found to be strongly correlated with daily milk-fat yield [17]. A recent study found that the abundance of Firmicutes in the rumen positively correlates with the average daily body weight gain in steers, suggesting that these bacteria play a significant role in feed efficiency of bovines [33]. Therefore, it is possible that the increase of Firmicutes to Bacteroidetes ratio induced by tannins can improve bovine performance, as previously suggested by other authors [19].

Fiber degradation is a complex process carried out by a group of microorganisms that are able to digest plant polysaccharides mainly through production of cellulolytic, hemicellulolytic, and pectinolytic enzymes [34]. Cellulolytic activity in the rumen involves a diverse bacterial community whose main members belong to genera* Ruminococcus* and* Fibrobacter*, while the main hemicellulolytic bacteria belong to genera* Ruminococcus*,* Prevotella,* and* Butyrivibrio *[34–37]. The blend of tannins administrated increased Ruminococcaceae and other members of phylum Firmicutes while inhibited genera* Prevotella* and* Fibrobacter*.* Prevotella* species have a documented role in metabolism of starch, hemicellulose, pectin, and protein catabolism [25]. The high abundance of this genus might be the result of a large metabolic niche that is occupied by different species with similar metabolic capabilities or it might be associated with a high genetic variability that enables members of genus* Prevotella* to occupy different ecological niches within the rumen [38–40]. For instance,* Prevotella* abundance has been shown to be higher in the rumen of animals of beef cattle with low feed efficiency [41]. Previous studies reported a great diversity in the sensitivity of* Prevotella* species to tannins [42], which could explain the lower abundance detected for this genus in tannins-supplemented steers. Since genus* Prevotella* is characterized by a high genetic diversity and wide array of output metabolites [38–40], their replacement by* Ruminococcus* may lead to a simpler product profile specialized to support the host's energy requirements [30]. Our results agree with previous studies that found a significant reduction in* Fibrobacter* and* Prevotella* rumen populations after supplementation with condensed and hydrolysable tannins, including chestnut and quebracho tannins [43, 44].

Rumen fibrolytic function is carried out by a largely redundant microbial community with overlapping distribution of metabolic capabilities, and this fibrolytic community has the ability to restore its structure and function after perturbation, a phenomenon known as* resilience* [27]. In this context, the observed changes in diversity of fibrolytic bacteria in tannins treated steers may result from a combination of physicochemical and biological mechanisms described in the literature, including direct interaction of tannins with fiber [45], which could alter the available surface area for microbial attack, the inhibition of certain fibrolytic taxa by means of tannins antimicrobial activity [46], and modulation of fibrolytic bacterial species driven by changes in rumen pH, since certain fibrolytic taxa of the rumen are inhibited at low pH under high-grain diets [36, 47].

Rumen amylolytic and saccharolytic bacteria were also affected by dietary treatment with tannins, mainly through the decrease of genera* Prevotella* and* Treponema*. Other amylolytic genera were moderately increased in tannins-fed steers including* Streptococcus*,* Bifidobacterium,* and* Lactobacillus*. Amylolytic activity is normally enhanced in ruminants consuming high-grain diets [18, 48, 49]. The rate of grain degradation by these microbial communities plays a key role in maintaining rumen homeostasis, since rapid starch fermentation produces large amounts of organic acids, therefore producing a drop in ruminal pH that may lead to metabolic acidosis [50]. A previous study showed that tannic acid and quebracho tannins lower the rate of microbial hydrolysis of starch-rich grains in the rumen by physical modification of the endosperm protein matrix [45]. This physical modulation of starch degradation could explain the lower abundance of sugar fermenting taxa detected in tannins treated steers, as well as the higher ruminal pH observed in this group.

Fermentation products of microbial activity in rumen, mainly short-chain volatile fatty acids, serve as a major source of energy for ruminants and have a direct impact on the physiological parameters of the animal and feed utilization efficiency [16]. Some members of family Veillonellaceae, which produce propionate as a major fermentation product and have been associated with lower methane emissions [51], were enhanced in tannins treated animals. Interestingly, some* Selenomonas* species can break tannin-protein complexes and use tannins as energy source [52, 53]. Therefore, the higher abundance of* Selenomonas* in tannins treated steers may be partly due to availability of tannins as direct energy source. Meanwhile, members of class Erysipelotrichi, which have been linked to beef cattle feed efficiency [33, 51, 54], were also modulated by tannins treatment.

Methane production during fermentation of feeds in the rumen represents a loss of 2–12% of gross energy [1], and it is performed by a group of archaea known collectively as methanogens which belong to phylum Euryarchaeota. The microorganisms produce methane, the second largest anthropogenic greenhouse gas which has a global warming potential 25 times that of carbon dioxide [55]. In the present study, a reduction of methanogenic archaea in tannins supplemented steers was detected as well as an inverse correlation between the abundances of phylum Euryarchaeota and rumen pH (*r* = −0.95). Tannins are thought to directly inhibit methanogens, as well as indirectly limit methanogenesis through reduction of hydrogen availability [1, 55]. Saminathan et al. found a significant decrease in genus* Methanobrevibacter* after treatment of rumen samples with condensed tannins* in vitro* [56]. Other authors described a linear decrease of genus* Methanobrevibacter* after dietary supplementation with condensed tannins from pine bark in goats [31].

Ruminal pH was significantly higher in tannins treated steers than in the control group, while urease activity exhibited the opposite pattern. These results agree with previous reports which found an increase in ruminal pH after supplementation with chestnut and quebracho tannins [57, 58]. Other authors observed that feeding chestnut and quebracho tannins decreased urease activity in the faeces of cows [10, 11].

Feed-grade urea is an effective source of nitrogen commonly used in beef cattle diets. Ureolytic bacteria in the rumen produce urease to hydrolyse urea to ammonia, which is subsequently used for the synthesis of amino acids and microbial protein. Normally, the rate of urea hydrolysis exceeds the rate of ammonia utilization, which leads to poor efficiency of urea utilization and increases toxic ammonia concentrations in blood [59]. Ureolytic bacteria in the rumen comprise a highly diverse group whose main species belong to genera* Succinivibrio*,* Treponema*,* Bacteroides*,* Butyrivibrio*,* Streptococcus,* and* Bifidobacterium* [60, 61]. We found that the most abundant ureolytic genera,* Butyrivibrio* and* Treponema*, were negatively affected by tannins treatment. Thus, the observed decline in ruminal urease activity may be related with the decrease of these urease-producing taxa. A previous study also observed a drop in ruminal urease activity after addition of tannins to diet but a direct interaction between tannins and urease enzyme was postulated as responsible for this inhibition [62].

## 5. Conclusions

The current study showed that chestnut and quebracho tannins added to the diet of Holstein steers modified rumen microbiota composition, particularly fiber and starch degrading bacteria, mainly by reducing the abundance of* Prevotella* and* Fibrobacter* while favoring Ruminococcaceae and other members of phylum Firmicutes. Tannins treatment significantly increased pH and decreased urease activity in the ruminal liquor. Further work is necessary to assess the possible relation between tannins ability to modify rumen microbiota composition and the alteration of rumen fermentation parameters associated with energy and feed efficiency of beef cattle, such as the profile of short-chain fatty acids and the emissions of ammonia and methane.

## Figures and Tables

**Table 1:** Formulation and composition of diet as percentage of dry matter.

Ingredients	% of DM
Alfalfa bale	19.0
Ground corn grain	64.0
Soybean meal	16.0
Trace mineral and vitamins	0.8
Tannins blend	0.2

*Total*	100.0

Composition analysis	% of DM

CP	15.0
RDP	9.3
Total calcium	0.9
Total phosphorus	0.4

Energy analysis	Mcal/kg

ME	2.97
NEm	2.01
NEg	1.35

DM: dry matter; CP: crude protein; RDP: rumen degradable protein; ME: metabolizable energy; NEm: net energy for maintenance; NEg: net energy for gain.
